# Supplementary material for: Electric Source Imaging in Presurgical Evaluation of Epilepsy: An Inter-Analyser Agreement Study
Source: Diagnostics (Basel). 2022 Sep 24;12(10):2303. doi: 10.3390/diagnostics12102303 (PMC9601236; doi:10.3390/diagnostics12102303)
Supplement: Supplementary file 1 [file diagnostics-12-02303-s001.zip › diagnostics-1847073-supplementary.pdf]

Supplementary Table S1. Sub-lobar regions. Each source was localised in one of the following sub-lobar regions. If the result of ESI showed multiple sources or was not clear, source location was defined as “non-localizable/diffuse” (if possible, right/left descriptors were added). If the source was located on the border of a clear lesion or cavity, the location was defined as “perilesional”

**Region**	**Side**	**Sub-region**
frontal	left/right	perisylvian-superior surface / operculum
		lateral
		mesial
		polar
		orbitofrontal
temporal	left/right	mesial
		polar
		basal
		lateral-anterior
		lateral-posterior
		perisylvian-inferior surface / opercular
central	left/right	lateral convexity
		mesial
		central sulcus – anterior surface
		central sulcus – posterior surface
		perisylvian-superior surface / opercular
parietal	left/right	lateral-convexity
		mesial
		perisylvian-superior surface / opercular
occipital	left/right	lateral
		mesial
		basal
insular	left/right	na
non-localizable / diffuse	left/right	na
non-localizable / diffuse	na	na
perilesional	na	na

Supplementary table legend 1. na: not applicable.
